# Distinct Liver Enzyme Profiles in Non-Diabetic Obesity: Associations with Obesity Severity and Metabolic Dysfunction

**DOI:** 10.3390/jcm15197341

**Published:** 2026-09-22

**Authors:** Muath Alsaidan, Daliyah M. Alruwaili, Tahani M. Alharthi, Faisal Alshuweishi, Bader F. Alshwmer, Yazeed Alshuweishi

**Affiliations:** 1 Department of Family and Community Medicine, College of Medicine, King Saud University, Riyadh 12372, Saudi Arabia; 2 Department of Clinical Laboratory Sciences, College of Applied Medical Sciences, King Saud University, Riyadh 12372, Saudi Arabia; 3 Department of Clinical Nutrition, King Khalid University Hospital (KKUH), King Saud University Medical City, Riyadh 12372, Saudi Arabia; 4 Department of Pathology and Laboratory Medicine, King Khalid University Hospital (KKUH), King Saud University Medical City, Riyadh 12372, Saudi Arabia

**Keywords:** obesity, metabolic dysfunction, gamma-glutamyl transferase (GGT), liver enzymes, cardiometabolic risk

## Abstract

**Background**: Obesity is a heterogeneous condition encompassing metabolically healthy and metabolically unhealthy phenotypes. Routine liver enzymes may reflect metabolic heterogeneity among individuals with obesity, yet their metabolic relevance in non-diabetic obesity remains incompletely characterized, particularly in Arab populations. This study examined the associations of routine liver enzymes with obesity severity and metabolic abnormalities in Saudi adults with non-diabetic obesity. **Methods**: This cross-sectional study included 168 non-diabetic adults with obesity. Alanine aminotransferase (ALT), aspartate aminotransferase (AST), gamma-glutamyl transferase (GGT), and alkaline phosphatase (ALP) were evaluated in relation to obesity severity, prediabetes, hypercholesterolemia, hypertriglyceridemia and metabolic syndrome (MetS). Correlation, multivariable linear regression, and receiver operating characteristic (ROC) analyses were performed to examine associations with obesity severity, prediabetes, hypercholesterolemia, and hypertriglyceridemia. **Results**: ALT, AST, and GGT generally increased with obesity severity, with ALT and GGT significantly higher in Class III than Class I obesity. GGT showed the most consistent differences across metabolic abnormalities, with higher concentrations in participants with prediabetes (20.0 vs. 16.0 U/L), hypercholesterolemia (21.0 vs. 16.0 U/L), and hypertriglyceridemia (22.0 vs. 17.0 U/L). GGT was also higher in participants with MetS (20.0 vs. 16.0 U/L). After multivariable adjustment, GGT remained positively associated with HbA1c (β = 0.031) and triglycerides (β = 0.188), while ALT was associated with BMI (β = 0.04). ROC analyses showed modest, outcome-specific discrimination, with GGT showing the highest discrimination for hypertriglyceridemia (AUC = 0.68). **Conclusions**: Liver enzyme profiles, particularly GGT and ALT, are associated with distinct dimensions of metabolic dysfunction in non-diabetic adults with obesity. GGT demonstrated the most consistent metabolic relationships with glycemic and lipid abnormalities, although the modest discriminatory performance supports its potential role as a complementary rather than standalone metabolic indicator.

## 1. Introduction

Obesity has become one of the most pressing public health challenges worldwide, affecting more than one billion individuals and contributing substantially to the global burden of chronic disease [1]. Although obesity is conventionally classified according to body mass index (BMI), BMI alone does not adequately distinguish individuals according to metabolic health [2,3]. This has led to the increasing recognition of distinct metabolic phenotypes, particularly metabolically healthy obesity (MHO) and metabolically unhealthy obesity (MUO) [4]. MHO generally describes individuals with obesity who have a relatively favorable metabolic profile, whereas MUO is characterized by metabolic abnormalities such as dysglycemia, dyslipidemia, hypertension, and insulin resistance [5,6,7,8]. However, definitions of MHO vary considerably across studies, and metabolic health in obesity should not necessarily be regarded as a permanent state. Individuals may transition from a metabolically favorable to an unfavorable phenotype over time, with important implications for future cardiovascular and metabolic risk [4]. Accordingly, contemporary approaches to obesity increasingly emphasize metabolic phenotype and cardiometabolic risk in addition to BMI-based classification.

The liver plays a central role in systemic glucose, lipid, and energy homeostasis [9,10,11]. In obesity, excessive nutrient availability and adipose tissue dysfunction promote ectopic lipid accumulation within hepatocytes, oxidative stress, mitochondrial dysfunction, chronic inflammation, and alterations in hepatocellular integrity [12,13]. These pathological processes may occur even before the development of clinically apparent liver disease or overt diabetes and are frequently reflected by subtle changes in circulating liver enzyme concentrations [14]. Elevated concentrations of routine liver enzymes, including alanine aminotransferase (ALT), aspartate aminotransferase (AST), alkaline phosphatase (ALP), and gamma-glutamyl transferase (GGT), are widely recognized as biomarkers of hepatocellular injury and are among the earliest biochemical indicators of MASLD [15,16]. Accumulating evidence indicates that liver enzymes are closely linked to metabolic dysfunction beyond their traditional role as markers of hepatic injury [17]. Elevated ALT and GGT have been associated with metabolic syndrome, insulin resistance, and the subsequent development of T2DM [18,19]. Although GGT is highly sensitive to hepatocellular injury, it is less specific than other liver enzymes because its concentrations may also be influenced by oxidative stress, inflammation, and hepatobiliary disorders [20]. These observations suggest that routinely available liver enzymes may provide metabolic information beyond their conventional role as markers of hepatic injury.

The distinction between MHO and MUO further highlights the need for accessible biomarkers that can characterize metabolic heterogeneity among individuals with obesity. Although individuals classified as metabolically healthy may have a more favorable cardiometabolic profile than those with MUO, MHO is not necessarily a stable or uniformly low-risk condition. Recent evidence indicates that transition toward an unhealthy metabolic phenotype is associated with increased cardiometabolic risk [21]. Routine biochemical markers that vary across obesity and metabolic phenotypes could therefore contribute to the characterization of metabolic health, although longitudinal studies are required to establish whether such markers predict subsequent metabolic deterioration.

Despite these associations, the metabolic significance of routine liver enzymes in adults with obesity who have not developed diabetes remains incompletely characterized, particularly in Arab populations, including Saudi Arabia. Previous studies have predominantly focused on individuals with established diabetes, metabolic syndrome, or Metabolic dysfunction-associated steatotic liver disease MASLD, in whom liver enzyme abnormalities may reflect more advanced metabolic impairment. Evidence characterizing liver enzyme patterns across different dimensions of metabolic dysfunction within non-diabetic Arab populations with obesity remains comparatively limited. Non-diabetic adults with obesity therefore represent an informative population for examining earlier metabolic alterations, with less influence from chronic hyperglycemia or glucose-lowering therapies. Characterizing liver enzyme patterns in this setting may provide insight into metabolic disturbances preceding overt diabetes and their potential utility as accessible indicators of cardiometabolic risk. Furthermore, the low prevalence of alcohol consumption in Saudi Arabia may reduce an important source of confounding when examining obesity-related liver enzyme alterations. Therefore, this study aimed to characterize the patterns of routinely measured liver enzymes (ALT, AST, GGT, and ALP) of Saudi adults with non-diabetic obesity and examine their relationships with obesity severity and metabolic dysfunction, including glycemic abnormalities, dyslipidemia, and metabolic syndrome. We further evaluated their independent associations with continuous metabolic markers and their discriminatory performance for obesity severity and metabolic abnormalities.

## 2. Methods

### 2.1. Study Design and Participant Selection

This retrospective, cross-sectional study was conducted using clinical data extracted from the family medicine clinic at King Khalid University Hospital, Riyadh, Saudi Arabia. The study protocol was reviewed and approved by the Institutional Review Board of King Saud University Medical City on 26 April 2026 (approval no. R26-IRB-288). The study was conducted in accordance with the Declaration of Helsinki. Because this retrospective study relied exclusively on de-identified clinical records, the requirement for individual informed consent was waived by the Institutional Review Board. The study was conducted and reported in accordance with the STROBE (Strengthening the Reporting of Observational Studies in Epidemiology) guidelines. The completed STROBE checklist is available in the Supplementary File (Table S1).

The master dataset comprised 218 records. Sequential exclusions were applied as follows: duplicate medical record numbers (*n* = 2), age younger than 18 years (*n* = 6), documented type 2 diabetes mellitus (*n* = 31), documented liver diseases (*n* = 7), and body mass index (BMI) below 30 kg/m^2^ (*n* = 4), yielding a final analytic cohort of 168 non-diabetic adults with obesity (Figure 1). A condition was considered documented only when an established clinical diagnosis had been recorded by a treating physician in the patient’s electronic medical record. After these sequential exclusions, the final analytic cohort comprised 168 adults with obesity and without documented type 2 diabetes mellitus.

### 2.2. Data Collection

Clinical data were retrieved from electronic health records and de-identified to ensure the protection of patient privacy and confidentiality. Anthropometric and demographic data were extracted from the clinical registry. Body weight and height were assessed using a calibrated weighing scale and a portable stadiometer (Marsden H226, Marsden Weighing Group, South Yorkshire, UK). Weight measurements were obtained while participants wore light clothing. Body mass index (BMI) was determined as weight in kilograms divided by height in meters squared (kg/m^2^). Participants’ medical histories and current medication use were also reviewed to confirm eligibility according to the predefined inclusion criteria.

### 2.3. Sample Collection and Biochemical Analysis

Laboratory data were retrieved from the clinical registry and included serum alanine aminotransferase (ALT), aspartate aminotransferase (AST), gamma-glutamyl transferase (GGT), alkaline phosphatase (ALP), glycated hemoglobin (HbA1c), total cholesterol (TC), triglycerides (TG), high-density lipoprotein cholesterol (HDL), and low-density lipoprotein cholesterol (LDL). All measurements had been performed during routine clinical assessment at the corresponding outpatient visits using standardized and validated laboratory procedures. Liver enzyme activities and lipid concentrations were analyzed using the Cobas 8000 automated platform (Roche Diagnostics, Rotkreuz, Switzerland), whereas HbA1c was determined using the Cobas 513 analyzer (Roche Diagnostics, Rotkreuz, Switzerland). Routine internal quality control and laboratory quality assurance procedures were implemented to ensure the reliability and consistency of analytical measurements.

### 2.4. Definitions

Obesity was classified according to body mass index (BMI) using established World Health Organization (WHO) and Saudi guidelines [22,23,24]. Class I obesity was defined as a BMI of 30.0–34.9 kg/m^2^, class II obesity as 35.0–39.9 kg/m^2^, and class III obesity as ≥40.0 kg/m^2^. For binary analyses, participants with class I or II obesity (BMI 30.0–39.9 kg/m^2^) were categorized as having non-severe obesity, whereas those with class III obesity (BMI ≥ 40.0 kg/m^2^) were classified as having severe obesity. Glycemic status was classified according to glycated hemoglobin (HbA1c) levels, consistent with the American Diabetes Association (ADA) and Saudi Ministry of Health criteria [25,26]. Normoglycemia was defined as HbA1c < 5.7% without the use of glucose-lowering medications, whereas prediabetes was defined as HbA1c ≥ 5.7%. Participants receiving metformin for prediabetes or polycystic ovarian syndrome (PCOS), in accordance with institutional clinical practice, were also included in the prediabetes group. Hypercholesterolemia (HC) was defined according to NCEP ATP III-based criteria as the presence of at least one of the following: total cholesterol (TC) ≥ 5.2 mmol/L, LDL ≥ 3.4 mmol/L or current use of lipid-lowering therapy [27,28]. Participants who did not meet any of these criteria were classified as having normal cholesterolemia (NC). Hypertriglyceridemia (HTG) was defined as a serum triglyceride concentration ≥ 1.69 mmol/L or current use of lipid-lowering therapy. Participants with TG < 1.69 mmol/L who were not receiving lipid-lowering therapy were classified as having normal triglyceride levels (NTG) [27,28]. The lipid-lowering medications recorded in the cohort were exclusively statins-atorvastatin, simvastatin, and rosuvastatin; no other classes of lipid-lowering medications were recorded. Metabolic syndrome was defined using a modified NCEP ATP III framework. Participants were classified as having metabolic syndrome when at least three of the following criteria were present: obesity (BMI ≥ 30 kg/m^2^), elevated triglycerides (≥1.7 mmol/L), reduced HDL (<1.0 mmol/L in men or <1.3 mmol/L in women), elevated blood pressure (≥130/85 mmHg or antihypertensive treatment), and impaired glycemic status (HbA1c ≥ 5.7% or glucose-lowering treatment) [29]. BMI and HbA1c were used in place of waist circumference and fasting plasma glucose, respectively, because these measurements were not available in the retrospective dataset [30,31]. Abnormal liver enzyme levels were defined using previously reported sex-specific upper reference limits [32,33]. Elevated alanine aminotransferase (ALT) was defined as >45 U/L in men and >34 U/L in women; elevated aspartate aminotransferase (AST) as >35 U/L in men and >31 U/L in women; elevated gamma-glutamyl transferase (GGT) as >55 U/L in men and >38 U/L in women; and elevated alkaline phosphatase (ALP) as >129 U/L in men and >104 U/L in women.

### 2.5. Statistical Analysis

Quantitative variables are presented as mean ± standard deviation (SD) or median with interquartile range (IQR; 25th–75th percentile), as appropriate. The distributions of liver enzyme levels were assessed using the Shapiro–Wilk test and were found to deviate significantly from normality (*p* < 0.05 for all enzymes). Accordingly, non-parametric tests were used for group comparisons. Differences in anthropometric and baseline characteristics between males and females were assessed using the Mann–Whitney U test. Comparisons across the three BMI classes were performed using the Kruskal–Wallis test, followed by Dunn’s post hoc test for pairwise comparisons when appropriate. Differences according to glycemic status, hypercholesterolemia, and hypertriglyceridemia status were evaluated using the Mann–Whitney U test.

Spearman correlation analysis was used to assess associations between liver markers and metabolic variables. Multivariable linear regression analyses were subsequently performed to evaluate the independent associations between liver enzyme levels and metabolic variables after adjustment for age, sex, smoking status, and previous bariatric surgery. Variables with non-normal distributions were log-transformed before inclusion in the regression models to improve normality and satisfy model assumptions. Regression results are reported as β coefficients with 95% confidence intervals (CIs) and corresponding *p* values.

All statistical analyses were performed using GraphPad Prism version 10.3.1 (GraphPad Software, Boston, MA, USA). All tests were two-tailed, and a *p* value < 0.05 was considered statistically significant.

## 3. Results

Baseline Clinical, Metabolic, and Liver Enzyme Characteristics of Adults with Obesity According to Sex:

A total of 168 non-diabetic adults with obesity were included in the analysis. Females constituted the majority of the study population 126 (75%), while males accounted for 42 (25%). Baseline clinical and biochemical characteristics stratified by sex are presented in Table 1. Compared with females, males had significantly higher SBP, DBP, TG, LDL, ALT, AST and GGT (all *p* < 0.05), whereas HDL was significantly higher in females (*p* = 0.021). No significant sex differences were observed for age, BMI, HbA1c, total cholesterol or ALP.

Additionally, the study included participants across all three obesity classes, with Class II obesity being the most common (41.7%), followed by Class III (34.5%) and Class I (23.8%). Prediabetes was present in 29.8% of participants, while hypercholesterolemia and hypertriglyceridemia were identified in 43.2% and 22.6%, respectively.

Liver Enzyme Profiles Vary with Obesity Severity and Metabolic Abnormalities, With GGT Showing the Most Consistent Differences:

The distribution of liver enzyme concentrations across obesity classes and metabolic subgroups is presented in Figure 2A–D. ALT concentrations increased with obesity severity, from a median of 13.4 U/L in Class I to 15.6 U/L in Class II and 19.8 U/L in Class III obesity, with significantly higher concentrations in Class III than in Class I (*p* = 0.004). GGT showed a similar pattern, increasing from 15.0 U/L in Class I to 18.0 U/L in Class II and 19.5 U/L in Class III, with a significant difference between Class III and Class I (*p* = 0.024). Although AST concentrations increased numerically across obesity classes (15.4, 16.0, and 17.3 U/L, respectively), the Class III versus Class I comparison did not reach statistical significance (*p* = 0.055). ALP did not differ significantly across obesity classes.

Across glycemic subgroups, participants with prediabetes had significantly higher median GGT concentrations than normoglycemic participants (20.0 vs. 16.0 U/L; *p* = 0.010), as well as higher ALP concentrations (84.5 vs. 71.0 U/L; *p* = 0.037). ALT and AST did not differ significantly between the glycemic groups.

Likewise, GGT concentrations were significantly higher in participants with hypercholesterolemia than in those with normal cholesterol levels (21.0 vs. 16.0 U/L; *p* = 0.0006), whereas ALT, AST, and ALP concentrations did not differ significantly between the groups (Figure 2).

Participants with hypertriglyceridemia had significantly higher median ALT (20.90 vs. 15.30 U/L, *p* = 0.028), AST (18.25 vs. 15.90 U/L, *p* = 0.023), and GGT (22.00 vs. 17.00 U/L, *p* < 0.001) concentrations than those with normal triglyceride levels. In contrast, ALP concentrations were comparable between the groups (75.00 vs. 77.00 U/L, *p* = 0.841).

GGT Shows the Broadest Associations with Metabolic Dysfunction Among Liver Enzymes:

Spearman’s correlation analysis (Table S2) identified GGT as the enzyme with the broadest pattern of significant correlations. GGT was positively correlated with BMI (r = +0.222, *p* = 0.005), with HbA1c (r = +0.243, *p* = 0.006), TG (r = +0.301, *p* < 0.001), and inversely correlated with HDL (r = −0.216, *p* = 0.011). ALT was positively correlated with BMI (r = +0.265, *p* < 0.001) and inversely correlated with HDL (r = −0.273, *p* = 0.001). AST showed a significant positive correlation with BMI only (r = +0.173, *p* = 0.031). ALP was inversely correlated with HDL (r = −0.226, *p* = 0.008) and positively correlated with HbA1c (r = +0.198, *p* = 0.026.

Moreover, multivariable linear regression analyses were performed to examine the independent associations between liver enzymes and continuous markers of metabolic dysfunction after adjustment for age, sex, smoking status, and previous bariatric surgery (Table 2). ALT was positively associated with BMI (β = 0.04, 95% CI: 0.01–0.08; *p* = 0.03). However, ALT was not significantly associated with HbA1c (β = 0.01, 95% CI: −0.01 to 0.04; *p* = 0.39), TG (β = 0.04, 95% CI: −0.09 to 0.18; *p* = 0.55), or HDL (β = −0.01, 95% CI: −0.08 to 0.04; *p* = 0.55). AST showed no significant associations with any of the investigated metabolic markers. Its association with BMI was positive but not statistically significant (β = 0.04, 95% CI: −0.01 to 0.10; *p* = 0.15). Similarly, AST was not associated with HbA1c (β = −0.01, 95% CI: −0.05 to 0.03; *p* = 0.54), TG (β = −0.05, 95% CI: −0.27 to 0.16; *p* = 0.63), or HDL (β = −0.02, 95% CI: −0.12 to 0.07; *p* = 0.59). In contrast, GGT demonstrated the broadest pattern of significant metabolic associations. Higher GGT concentrations were positively associated with HbA1c (β = 0.031, 95% CI: 0.01–0.06; *p* = 0.01) and TG (β = 0.188, 95% CI: 0.07–0.30; *p* = 0.01). The association between GGT and BMI was positive but did not reach statistical significance (β = 0.02, 95% CI: −0.01 to 0.06; *p* = 0.11), while its inverse association with HDL was also non-significant (β = −0.04, 95% CI: −0.09 to 0.01; *p* = 0.13). Moreover, higher ALP concentrations were significantly associated with lower HDL concentrations (β = −0.11, 95% CI: −0.23 to −0.01; *p* = 0.04). A positive association was also observed between ALP and TG, although it did not reach statistical significance (β = 0.237, 95% CI: −0.01 to 0.48; *p* = 0.06). No significant associations were observed between ALP and BMI (β = 0.02, 95% CI: −0.05 to 0.09; *p* = 0.53) or HbA1c (β = −0.018, 95% CI: −0.07 to 0.03; *p* = 0.48).

Liver Enzymes Show Modest and Outcome-Specific Discrimination of Metabolic Abnormalities:

ROC curve analysis showed limited-to-modest discriminatory performance of the liver enzymes for the evaluated metabolic abnormalities (Table 3). For prediabetes, GGT and ALP showed significant discrimination, whereas ALT and AST did not. GGT was the only liver enzyme showing significant discrimination for hypercholesterolemia and hypertriglyceridemia. The highest AUC was observed for GGT in relation to hypertriglyceridemia (AUC = 0.68, 95% CI: 0.58–0.78; *p* = 0.001).

Higher Liver Enzyme Levels Are Observed in Adults With Obesity and Metabolic Syndrome:

As shown in Figure 3, subjects with metabolic syndrome (+MetS) exhibited higher liver enzyme levels than those without metabolic syndrome (−MetS). ALT showed a marked elevation in the +MetS group, with a median of 20.00 U/L compared with 14.35 U/L in the −MetS group (*p* = 0.0034). GGT was similarly higher in Subjects with +MetS (20.00 vs. 16.00 U/L, *p* = 0.0032), while AST showed a smaller but significant difference (17.55 vs. 15.10 U/L, *p* = 0.0211). ALP tended to be higher in the +MetS group (84.50 vs. 71.50 U/L), but this difference was borderline and did not meet the conventional threshold for statistical significance (*p* = 0.0560). Consistent with these group differences, ROC analysis suggested modest discriminatory ability of the liver enzymes for MetS status.

## 4. Discussion

In this study of non-diabetic adults with obesity, liver enzyme profiles showed distinct relationships with obesity severity and metabolic dysfunction, with GGT emerging as the most consistent marker of an unfavorable metabolic phenotype. ALT was strongly related to obesity severity and was also higher among participants with hypertriglyceridemia. AST showed fewer associations overall but was likewise higher in the hypertriglyceridemia group, whereas ALP demonstrated comparatively less consistent associations. Notably, GGT was consistently higher across several metabolic abnormalities, including prediabetes, hypercholesterolemia, hypertriglyceridemia, and metabolic syndrome, and showed the broadest correlations with adiposity, glycemic, and lipid measures. These relationships were partly retained after multivariable adjustment, with GGT independently associated with HbA1c and triglycerides, whereas ALT was primarily associated with BMI. ROC analyses further supported these differential patterns, with GGT providing the most consistent, albeit modest, discrimination across the evaluated metabolic abnormalities. It is important to note that median liver enzyme concentrations generally remained within conventional reference ranges, significant variations were observed across obesity and metabolic phenotypes. These differences may reflect subtle metabolic alterations rather than overt hepatocellular injury. Together, these findings suggest that alterations in routinely measured liver enzymes, particularly GGT and ALT, may reflect different dimensions of metabolic dysfunction in obesity even in the absence of established diabetes, while their modest discriminatory performance underscores their potential value as complementary rather than standalone metabolic indicators.

Previous studies have consistently demonstrated that liver enzyme concentrations are elevated in individuals with obesity and metabolic syndrome compared with metabolically healthy populations. For example, a cross-sectional study of adult women reported significantly higher ALT, ALP, and GGT concentrations in obese individuals than in non-obese controls, whereas AST showed no significant difference, highlighting the differential responses of individual liver enzymes to obesity [34]. Similarly, a recent systematic review and meta-analysis including more than 278,000 participants demonstrated significantly higher ALT, AST, and GGT concentrations in individuals with metabolic syndrome, with GGT exhibiting the largest difference between affected and unaffected individuals [35]. However, these studies primarily evaluated obesity or metabolic syndrome as binary outcomes and provided limited insight into whether routine liver enzymes differentially reflect specific manifestations of metabolic dysfunction within an already obese population. In contrast, the present study focused exclusively on adults with non-diabetic obesity and examined individual components of metabolic dysfunction, including severe obesity, prediabetes, hypercholesterolemia, and hypertriglyceridemia. We demonstrated that liver enzymes exhibit distinct metabolic profiles rather than changing uniformly across these outcomes. Specifically, GGT showed the strongest associations with dyslipidemia and prediabetes, whereas ALT was more closely associated with obesity severity, while AST and ALP demonstrated comparatively limited metabolic relevance. These findings may also be considered within the broader framework of metabolically healthy obesity (MHO) and metabolically unhealthy obesity (MUO). Differences in liver enzyme profiles across metabolic abnormalities, despite generally preserved concentrations, further highlight the metabolic heterogeneity of obesity. However, the absence of metabolic syndrome should not be considered equivalent to MHO, which requires specific prespecified criteria. Accordingly, our findings reflect variation across dimensions of metabolic dysfunction rather than a direct MHO-MUO comparison. Prospective studies using prespecified MHO/MUO definitions are needed to determine whether changes in routine liver enzymes accompany or predict metabolic deterioration.

Among the evaluated liver enzymes, GGT showed the most consistent associations with metabolic dysfunction, suggesting that its elevation may reflect broader cardiometabolic disturbances beyond hepatobiliary injury alone. In our cohort, GGT was associated with hypercholesterolemia, hypertriglyceridemia, and prediabetes, and its associations with HbA1c and triglycerides remained significant after adjustment for important clinical confounders. This pattern is biologically plausible given the broader involvement of GGT in cardiometabolic processes [36]. In addition to its role in glutathione metabolism and redox homeostasis, elevated GGT has been linked to oxidative stress, low-grade inflammation, insulin resistance, altered lipid metabolism, and vascular dysfunction, processes that frequently accompany obesity and metabolic dysfunction [37,38]. Circulating GGT is also heterogeneous and can be separated into four fractions—b-GGT (big), m-GGT (medium), s-GGT (small), and f-GGT (free)—which appear to exhibit distinct metabolic and cardiovascular associations. Notably, b-GGT and f-GGT have shown particularly strong relationships with BMI, glucose, triglycerides, insulin, and other features of metabolic syndrome, while b-GGT has also been linked to hepatic steatosis and identified within atherosclerotic plaques [39,40]. These observations further support the concept that circulating GGT may reflect a combination of hepatic, metabolic, inflammatory, and vascular processes.

Our findings are consistent with studies across different populations. A large study of Chinese adults demonstrated a progressive increase in the prevalence of hypertriglyceridemia, hypercholesterolemia, and low HDL across increasing serum GGT concentrations, with the strongest association observed for hypertriglyceridemia, indicating that GGT is particularly sensitive to disturbances in triglyceride metabolism [41]. Similarly, among non-diabetic adults with normal fasting triglyceride concentrations, GGT was the only routine liver enzyme independently associated with postprandial hypertriglyceridemia, whereas ALT, AST, and ALP showed no independent associations [42]. The close relationship between GGT and broader metabolic dysfunction has also been demonstrated in studies evaluating metabolic syndrome among young population. In adolescents, higher serum GGT concentrations were independently associated with metabolic syndrome after adjustment for established risk factors, with central obesity exhibiting the strongest relationship [43]. Likewise, a recent pediatric study reported that elevated GGT concentrations were associated with central adiposity, adverse lipid profiles, and an increased probability of metabolic syndrome, even during childhood [44]. Collectively, these findings are consistent with our observation that GGT shows broader associations with metabolic abnormalities than the other routinely measured liver enzymes. Nevertheless, GGT remains a nonspecific biomarker, and its associations with oxidative, inflammatory, hepatic, and vascular pathways should not be interpreted as evidence that GGT directly mediates these processes.

Unlike GGT, which demonstrated broad associations with multiple components of metabolic dysfunction, ALT appeared to be more closely related to obesity severity than to systemic metabolic abnormalities. Our findings are consistent with recent studies evaluating liver enzymes across obesity phenotypes. A large study of adults reported progressive increases in ALT, AST, and GGT with increasing BMI, with ALT showing the strongest association with obesity, whereas AST demonstrated weaker associations [45]. Similarly, another study found that ALT and GGT were independently associated with general and abdominal obesity, while AST showed substantially weaker associations [46]. Moreover, obesity-induced hepatic lipid accumulation, driven largely by visceral adiposity and increased free fatty acid flux to the liver, has been identified as a major determinant of ALT elevation, providing a biological explanation for its stronger association with obesity severity than with dyslipidemia [33]. In contrast, among metabolically healthy and unhealthy obesity phenotypes, both ALT and GGT increased with worsening metabolic status; however, GGT exhibited stronger associations with adverse cardiometabolic characteristics, whereas AST and ALP showed comparatively limited discriminatory value [47]. Collectively, these findings closely parallel our results, supporting the concept that ALT predominantly reflects obesity severity and hepatic steatosis, whereas GGT is more closely associated with the broader metabolic disturbances accompanying obesity.

An important implication of our findings is that routine liver enzymes should be regarded as complementary rather than interchangeable biomarkers of obesity-related metabolic dysfunction. Although all four enzymes are routinely included in liver function testing, they demonstrated distinct metabolic profiles in the present study, suggesting that each reflects different aspects of obesity-related pathophysiology. These observations are consistent with a recent comprehensive review highlighting that abnormalities in liver function tests among individuals with obesity are driven by multiple interrelated mechanisms, including hepatic steatosis, insulin resistance, oxidative stress, chronic low-grade inflammation, mitochondrial dysfunction, and adipose tissue dysfunction rather than by liver injury alone [48]. Consequently, individual liver enzymes likely reflect different biological pathways underlying obesity-related metabolic deterioration. Our findings therefore support the interpretation of routine liver enzymes as complementary biomarkers that provide distinct, rather than redundant, information regarding metabolic health in adults with non-diabetic obesity.

This study has several strengths. First, it comprehensively evaluated obesity-related metabolic dysfunction by integrating obesity severity, glycemic status, lipid abnormalities, and the four routinely measured liver enzymes (ALT, AST, GGT, and ALP), enabling a direct comparison of their metabolic profiles within the same cohort. Second, by focusing exclusively on adults with non-diabetic obesity, the study characterized early metabolic dysfunction while minimizing the confounding effects of established diabetes and diabetic-related complications on liver enzyme concentrations. Nevertheless, several limitations should be considered. First, the retrospective cross-sectional design precludes assessment of temporality or causality. Although participants with documented liver diseases were excluded, systematic liver imaging, elastography, or histological assessment was unavailable; therefore, undiagnosed hepatic steatosis and its severity could not be assessed. Second, BMI was the principal measure of adiposity, while waist circumference, body composition, and body-fat percentage were unavailable, limiting assessment of central and overall adiposity. Although previous bariatric surgery was included as a covariate in multivariable analyses, residual confounding remains possible. Potentially hepatotoxic medications were not a predefined exclusion criterion, further introducing potential medication-related confounding. Third, the predominantly female sample may limit generalizability and precluded adequately powered sex-stratified analyses, although sex was included as a covariate in multivariable models. Finally, the relatively modest sample size limited statistical precision and subgroup analyses. Prospective studies incorporating liver imaging, body-composition measures, fasting metabolic parameters, and more homogeneous populations are needed to validate these findings and assess the additional value of composite hepatic and metabolic indices.

## 5. Conclusions

Routine liver enzymes exhibit distinct metabolic profiles in adults with non-diabetic obesity. GGT showed the most consistent associations with dyslipidemia and glycemic abnormalities, whereas ALT was particularly associated with obesity severity and was also higher in participants with hypertriglyceridemia. AST showed fewer associations overall, while ALP demonstrated comparatively less consistent relationships with the metabolic characteristics examined. These findings suggest that routine liver enzymes may provide complementary information regarding metabolic heterogeneity in obesity; however, their modest discriminatory performance does not support their use as standalone indicators of metabolic risk. Larger prospective, multicenter studies are warranted to validate these findings and determine their prognostic relevance.

## Figures and Tables

**Figure 1:**
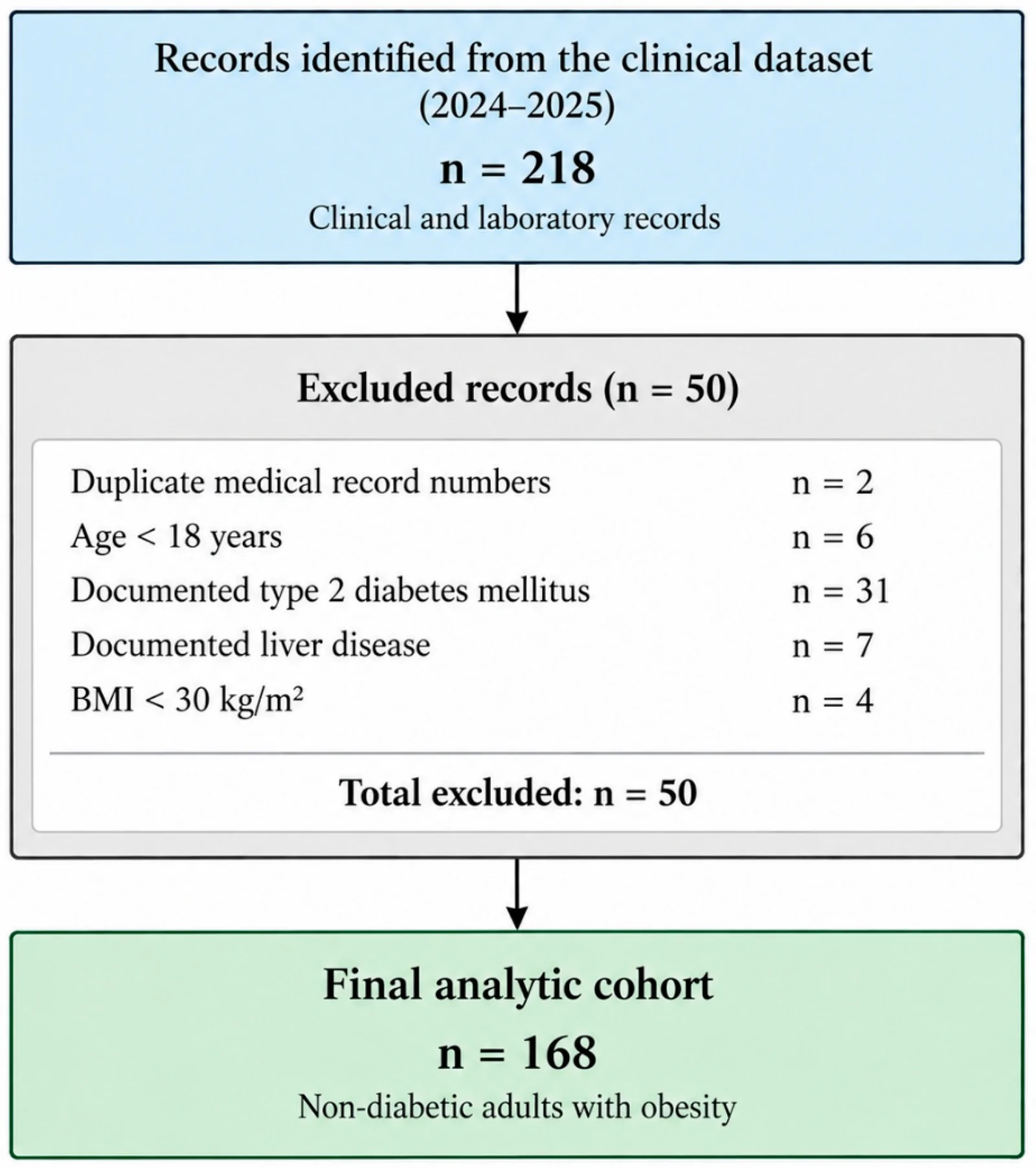
Participant-selection flow diagram.

**Figure 2:**
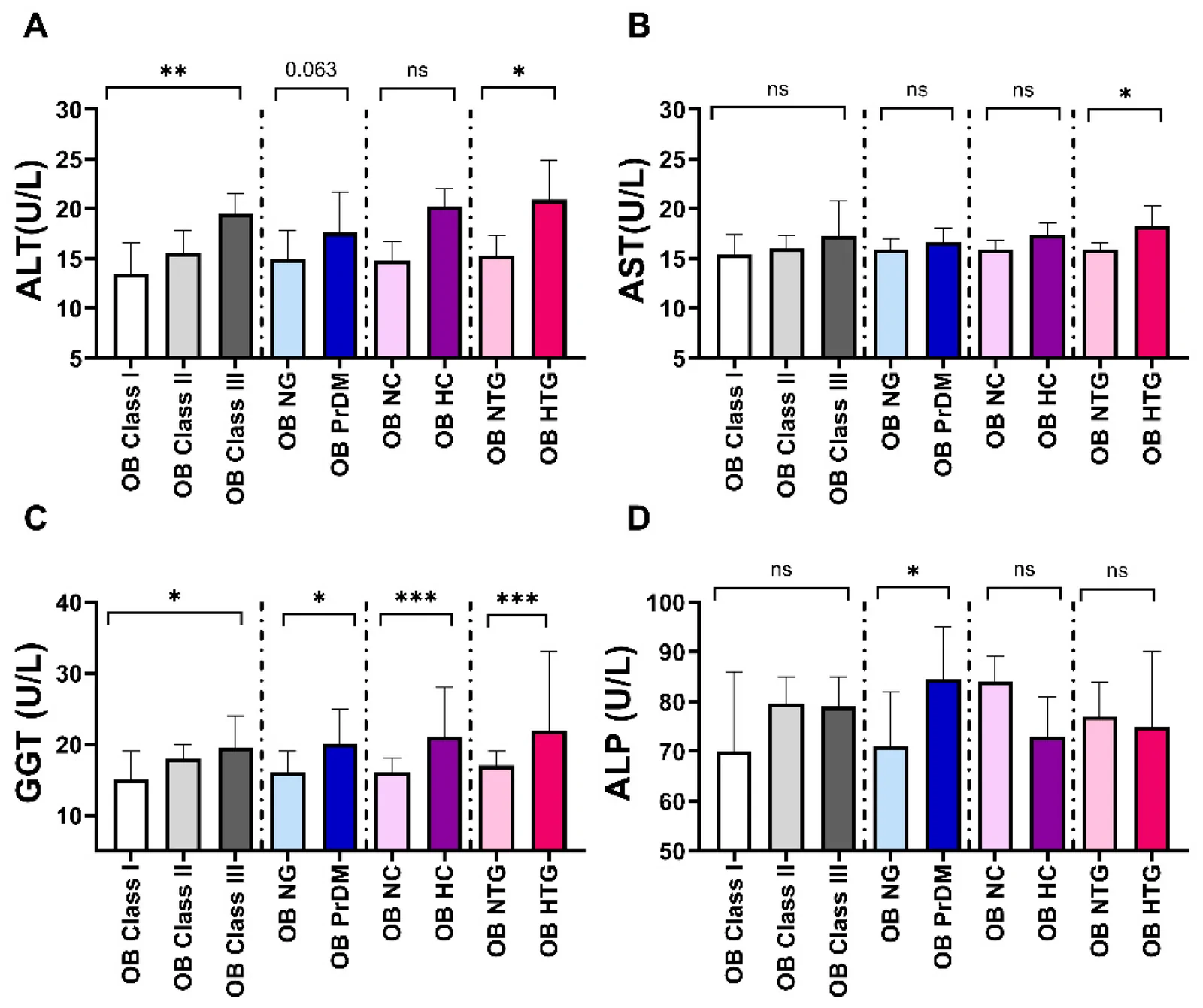
Distribution of liver enzyme concentrations according to obesity severity and metabolic abnormalities in obese non-diabetic adults. Bar charts illustrate the median concentrations of (**A**) alanine aminotransferase (ALT), (**B**) aspartate aminotransferase (AST), (**C**) gamma-glutamyl transferase (GGT), (**D**) alkaline phosphatase (ALP), across obesity classes and metabolic subgroups. For each enzyme, the first three bars represent Class I, Class II, and Class III obesity, followed by comparisons between normoglycemia (NG) and prediabetes (PreDM), normocholesterolemia (NC) and hypercholesterolemia (HC), and normal triglycerides (NTG) and hypertriglyceridemia (HTG). Abbreviations: ALT, alanine aminotransferase; AST, aspartate aminotransferase; GGT, gamma-glutamyl transferase; ALP, alkaline phosphatase; PreDM, prediabetes; HC, hypercholesterolemia; HTG, hypertriglyceridemia; ns, no statistical significance (*p* ≥  0.05); *, *p*  <  0.05; **, *p* < 0.01; ***, *p* < 0.001.

**Figure 3:**
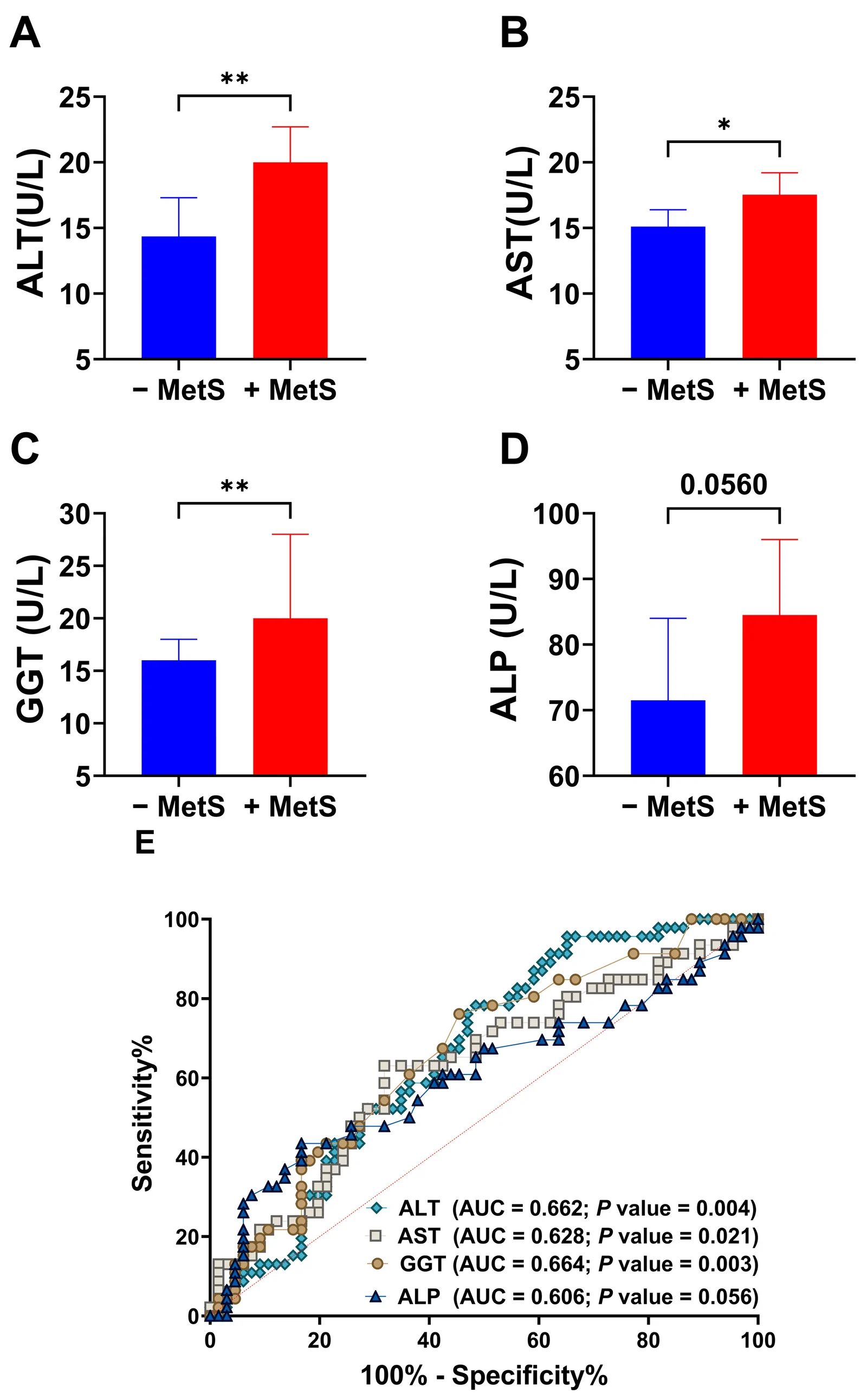
Liver enzyme profiles and ROC curve analyses according to metabolic syndrome status in adults with obesity. Median concentrations of (**A**) alanine aminotransferase (ALT), (**B**) aspartate aminotransferase (AST), (**C**) gamma-glutamyl transferase (GGT), and (**D**) alkaline phosphatase (ALP) in participants without metabolic syndrome (−MetS) and with metabolic syndrome (+MetS). (**E**) Receiver operating characteristic (ROC) curve analysis of ALT, AST, GGT, and ALP for discriminating participants with and without MetS. Bars represent median values with interquartile ranges. Group comparisons were performed using the Mann–Whitney U test. * denotes *p* < 0.05; ** denotes *p* < 0.01. Exact *p* values are shown where applicable. AUC, area under the receiver operating characteristic curve; ns, not significant.

**Table 1:** Biochemical Characteristics of Non-Diabetic Obese Individuals Stratified by Sex.

Variable	Male (n = 42)	Female (n = 126)	*p* Value
Age (years)	37.00 (31.25–43.00)	41.00 (33.50–46.00)	0.075
BMI (kg/m^2^)	39.30 (36.03–43.09)	37.74 (35.00–41.55)	0.211
SBP (mmHg)	134.50 (122.75–141.25)	125.00 (112.00–134.00)	0.001
DBP (mmHg)	75.00 (69.75–81.25)	70.00 (62.75–78.25)	0.008
HbA1c (%)	5.50 (5.25–5.90)	5.40 (5.20–5.70)	0.157
Total cholesterol (mmol/L)	5.20 (4.44–5.81)	4.83 (4.29–5.38)	0.080
TG (mmol/L)	1.16 (0.91–1.58)	0.94 (0.78–1.33)	0.026
HDL (mmol/L)	1.24 (1.13–1.42)	1.34 (1.21–1.57)	0.021
LDL (mmol/L)	3.25 (2.61–3.74)	2.88 (2.40–3.36)	0.032
ALT (U/L)	23.30 (17.60–26.60)	14.00 (10.97–20.00)	<0.001
AST (U/L)	18.50 (15.90–22.50)	15.10 (12.97–18.60)	<0.001
GGT (U/L)	28.00 (19.00–37.00)	16.00 (12.00–20.00)	<0.001
ALP (U/L)	81.00 (67.00–98.00)	75.00 (64.00–90.25)	0.480

Data are presented as Median (IQR: 25th–75th percentile). *p* values from the Mann–Whitney U test (exact method). Highlighted rows: *p* < 0.05. Abbreviations: BMI, body mass index; SBP, systolic blood pressure; DBP, diastolic blood pressure; TG, triglycerides; HDL, high-density lipoprotein; LDL, low-density lipoprotein; ALT, alanine aminotransferase; AST, aspartate aminotransferase; GGT, gamma-glutamyl transferase; ALP, alkaline phosphatase.

**Table 2:** Multivariable Linear Regression Analysis of Associations Between Liver Enzymes and Continuous Metabolic Markers.

Liver Enzyme	BMI β (95% CI); *p*	HbA1c β (95% CI); *p*	TG β (95% CI); *p*	TC β (95% CI); *p*	LDL β (95% CI); *p*	HDL β (95% CI); *p*
ALT	0.04 (0.01 to 0.08); 0.030	0.01 (−0.01 to 0.04); 0.390	0.04 (−0.09 to 0.18); 0.550	0.03 (−0.03 to 0.08); 0.331	0.02 (−0.07 to 0.11); 0.718	−0.01 (−0.08 to 0.04); 0.550
AST	0.04 (−0.01 to 0.10); 0.150	−0.01 (−0.05 to 0.03); 0.540	−0.05 (−0.27 to 0.16); 0.630	−0.05 (−0.14 to 0.03); 0.231	−0.09 (−0.23 to 0.05); 0.187	−0.02 (−0.12 to 0.07); 0.590
GGT	0.02 (−0.01 to 0.06); 0.110	0.03 (0.01 to 0.06); 0.010	0.19 (0.07 to 0.30); 0.010	0.05 (−0.0004 to 0.09); 0.052	0.04 (−0.04 to 0.12); 0.299	−0.04 (−0.09 to 0.01); 0.130
ALP	0.02 (−0.05 to 0.09); 0.530	−0.02 (−0.07 to 0.03); 0.480	0.24 (−0.01 to 0.48); 0.060	−0.04 (−0.14 to 0.06); 0.423	−0.10 (−0.26 to 0.06); 0.239	−0.11 (−0.23 to −0.01); 0.040

Abbreviations: ALT, alanine aminotransferase; AST, aspartate aminotransferase; GGT, gamma-glutamyl transferase; ALP, alkaline phosphatase; BMI, body mass index; HbA1c, glycated hemoglobin; TG, triglycerides; HDL, high-density lipoprotein cholesterol; TC, total cholesterol; LDL, low-density lipoprotein cholesterol; β, regression coefficient; CI, confidence interval. Footnote: Values are presented as adjusted regression coefficients (β) with 95% confidence intervals (CIs) and *p* values from separate multivariable linear regression models adjusted for age, sex, smoking status, and previous bariatric surgery. Liver enzyme concentrations and continuous metabolic variables were log-transformed before analysis.

**Table 3:** Receiver operating characteristic (ROC) curve analysis of liver enzymes for metabolic abnormalities among non-diabetic adults with obesity.

Liver Enzyme	PreDM AUC (95% CI)	*p*	HC AUC (95% CI)	*p*	HTG AUC (95% CI)	*p*
ALT	0.60 (0.50–0.70)	0.063	0.58 (0.49–0.68)	0.084	0.62 (0.51–0.73)	0.028
AST	0.52 (0.42–0.62)	0.702	0.57 (0.48–0.67)	0.135	0.63 (0.52–0.71)	0.023
GGT	0.64 (0.54–0.73)	0.011	0.67 (0.58–0.76)	<0.001	0.68 (0.58–0.78)	0.001
ALP	0.61 (0.51–0.71)	0.038	0.56 (0.47–0.66)	0.200	0.51 (0.39–0.63)	0.839

Abbreviations: ALT, alanine aminotransferase; AST, aspartate aminotransferase; GGT, gamma-glutamyl transferase; ALP, alkaline phosphatase; PreDM, prediabetes; HC, hypercholesterolemia; HTG, hypertriglyceridemia; AUC, area under the receiver operating characteristic curve; CI, confidence interval. Footnotes: Data are presented as the area under the receiver operating characteristic curve (AUC) with 95% confidence intervals (CI). An AUC of 0.50 indicates no discriminatory ability, 0.60–0.69 indicates poor-to-fair discrimination, 0.70–0.79 indicates acceptable discrimination, and ≥0.80 indicates good discrimination.

## Data Availability

The data that support the findings of this study can be provided by the corresponding author following a reasonable request and clearance from the primary institution.

## Supplementary Materials

The following supporting information can be downloaded at https://www.mdpi.com/article/10.3390/jcm15197341/s1, Table S1: STROBE Statement—Checklist of Items Reported for the Present Cross-Sectional Study. Table S2: Spearman’s Correlation Coefficients Between Liver Enzymes and Metabolic Variables.
