# Supplementary material for: Distinct Liver Enzyme Profiles in Non-Diabetic Obesity: Associations with Obesity Severity and Metabolic Dysfunction
Source: J Clin Med. 2026 Sep 22;15(19):7341. doi: 10.3390/jcm15197341 (PMC13665482; doi:10.3390/jcm15197341)
Supplement: Supplementary file 1 [file jcm-15-07341-s001.zip › jcm-4522708-supplementary.pdf]

Table S1. STROBE Statement—Checklist of Items Reported for the Present Cross-Sectional Study

Item No.	STROBE recommendation	Location in manuscript / information reported
1a	Indicate the study design with a commonly used term in the title or abstract.	Abstract, p. 1. The study is identified as a cross-sectional study.
1b	Provide an informative and balanced summary of what was done and what was found.	Abstract, pp. 1–2. Population, liver enzymes, statistical analyses, major findings, and conclusions are summarized.
2	Explain the scientific background and rationale for the investigation.	Introduction, pp. 2–3. Background on obesity heterogeneity, MHO/MUO, metabolic dysfunction, and liver enzymes is presented.
3	State specific objectives, including prespecified hypotheses where applicable.	Introduction, p. 3. Objectives include characterizing liver-enzyme profiles and examining their associations with obesity severity and metabolic dysfunction.
4	Present key elements of the study design early in the paper.	Methods, Section 2.1, p. 3. Retrospective cross-sectional design is explicitly stated.
5	Describe the setting, locations, and relevant dates, including periods of recruitment/exposure/data collection.	Methods, Section 2.1. Family medicine clinic, King Khalid University Hospital, Riyadh, Saudi Arabia. Clinical and laboratory records from 2024–2025 were reviewed.
6a	Give eligibility criteria and the sources and methods of participant selection.	Methods, Section 2.1, pp. 3–4. The master dataset contained 218 records; sequential exclusions yielded 168 eligible participants.
6b	For matched studies, give matching criteria and numbers in each group.	Not applicable. No matching was performed.
7	Clearly define all outcomes, exposures, predictors, potential confounders, and effect modifiers.	Methods, Sections 2.2–2.4. Obesity classes, glycemic status, hypercholesterolemia, hypertriglyceridemia, MetS, liver enzymes, and relevant covariates are defined.
8	Give sources of data and details of methods of assessment/measurement for each variable of interest.	Methods, Sections 2.2–2.3, pp. 4–5. Electronic medical records/clinical registry, anthropometric measurements, laboratory platforms, and quality-control procedures are described.

Item No.	STROBE recommendation	Location in manuscript / information reported
9	Describe any efforts to address potential sources of bias.	Methods/Discussion. Predefined eligibility criteria were applied consistently and standardized clinical/laboratory records were used. Potential medication-related, selection, and residual confounding are acknowledged in the limitations.
10	Explain how the study size was arrived at.	Methods, Section 2.1. "The study sample comprised all eligible records available during the predefined study period; therefore, no a priori sample-size calculation was performed."
11	Explain how quantitative variables were handled in the analyses and describe grouping choices.	Methods, Sections 2.4–2.5. BMI classes and biochemical cutoffs are defined; continuous variables and log transformations are described.
12a	Describe all statistical methods, including those used to control for confounding.	Methods, Section 2.5. Mann–Whitney U, Kruskal–Wallis with Dunn's post hoc test, Spearman correlation, multivariable linear regression, ROC analysis, and covariate adjustment are described.
12b	Describe methods used to examine subgroups and interactions.	Methods/Results. Analyses were conducted according to obesity class, glycemic status, cholesterol status, triglyceride status, and metabolic syndrome status.
12c	Explain how missing data were addressed.	Methods, Statistical Analysis. "Analyses were conducted using available complete data for each variable, and no imputation of missing values was performed."
12d	If applicable, describe analytical methods taking account of sampling strategy.	Not applicable. No complex sampling design was used.
12e	Describe any sensitivity analyses.	Not applicable / none performed.
13a	Report numbers of individuals at each stage of the study.	Methods, Section 2.1; Figure S1. Initial dataset n=218 and final analytic cohort n=168 are reported.
13b	Give reasons for non-participation/exclusion at each stage.	Methods, Section 2.1. Duplicate records (n=2), age <18 years (n=6), documented T2DM (n=31), documented liver disease (n=7), and BMI <30 kg/m² (n=4).
14a	Give characteristics of study participants and information on exposures and potential confounders.	Results, Table 1, p. 6. Demographic, anthropometric, metabolic, and liver-enzyme characteristics are reported.

Item No.	STROBE recommendation	Location in manuscript / information reported
14b	Indicate number of participants with missing data for each variable of interest.	Methods/Results. Available-case analysis was used; no imputation was performed. Variable-specific denominators should be shown where relevant in tables/analyses.
14c	Summarize follow-up time.	Not applicable. Cross-sectional study.
15	Report numbers of outcome events or summary measures.	Results, Tables 1–3 and Figures 1–2. Median liver-enzyme concentrations and subgroup comparisons are reported.
16a	Give unadjusted estimates and, if applicable, confounder-adjusted estimates with precision.	Results, Table 2. Adjusted β coefficients with 95% CIs and P values are reported; covariates are specified.
16b	Report category boundaries when continuous variables are categorized.	Methods, Section 2.4. Thresholds for BMI classes, HbA1c, lipids, BP, and liver-enzyme elevations are provided.
16c	If relevant, translate estimates of relative risk into absolute risk.	Not applicable. Relative-risk estimates were not used.
17	Report other analyses, including subgroup and sensitivity analyses.	Results. Obesity-class, metabolic-subgroup, MetS, correlation, multivariable regression, and exploratory ROC analyses are presented.
18	Summarize key results with reference to study objectives.	Discussion, opening paragraphs. Main findings for GGT, ALT, AST, and ALP are summarized in relation to obesity severity and metabolic dysfunction.
19	Discuss limitations, taking into account sources of potential bias or imprecision.	Discussion, pp. 13–14. Cross-sectional design, lack of imaging, absence of waist/body-composition measures, previous bariatric surgery, medication-related confounding, female predominance, and sample size are discussed.
20	Give a cautious overall interpretation considering objectives, limitations, multiplicity of analyses, and evidence from similar studies.	Discussion and Conclusion. Findings are interpreted as complementary metabolic associations rather than evidence of causality or standalone diagnostic utility.
21	Discuss generalisability of the study findings.	Discussion, Limitations. The single-center Saudi setting and predominantly female sample are acknowledged as factors that may limit generalisability.

Item No.	STROBE recommendation	Location in manuscript / information reported
22	Give the source of funding and the role of funders.	Funding statement, p. 14. Ongoing Research Funding Program (ORF-2026-1126), King Saud University.

Table Supplementary S2. Spearman's Correlation Coefficients Between Liver Enzymes and Metabolic Variables.

Variable	ALT		AST		GGT		ALP	
	r	*P*	r	*P*	r	*P*	r	*P*
BMI	+0.265	<0.001	+0.173	0.031	+0.222	0.005	+0.082	0.309
Total cholesterol	+0.054	0.531	−0.063	0.460	+0.165	0.053	−0.089	0.301
TG	+0.159	0.062	−0.014	0.873	+0.301	<0.001	+0.164	0.054
HDL-C	−0.273	0.001	−0.078	0.363	−0.216	0.011	−0.226	0.008
LDL-C	+0.045	0.605	−0.089	0.303	+0.113	0.188	−0.098	0.253
HbA1c	+0.135	0.129	−0.069	0.439	+0.243	0.006	+0.198	0.026

Abbreviations: TG, triglycerides; HDL, high-density lipoprotein; LDL, low-density lipoprotein; HbA1c, glycated haemoglobin. Footnote: Values are presented as Spearman's rank correlation coefficients (r) with corresponding two-tailed P values. Positive coefficients indicate direct correlations, whereas negative coefficients indicate inverse correlations. Statistical significance was defined as P < 0.05.
